# The Impact of Telenursing on Patients With Myocardial Infarction Based on a Self‐Care Program: A Randomized Clinical Trial

**DOI:** 10.1002/hsr2.73110

**Published:** 2026-08-26

**Authors:** Arian Mobasheri, Parand Pourghane, Fereshteh Besharati, Saman Maroufizadeh

**Affiliations:** ^1^ Zeynab (P.B.U.H) School of Nursing and Midwifery Guilan University of Medical Sciences Rasht Iran; ^2^ Department of Biostatistics and Epidemiology, School of Health Guilan University of Medical Sciences Rasht Iran

**Keywords:** medication adherence, myocardial infarction, self‐care, self‐efficacy, telemedicine, telenursing

## Abstract

**Background and Aims:**

Self‐care and medication adherence are key factors in the management of patients with myocardial infarction (MI), but many patients face challenges in these areas and require novel care approaches. This study, based on a self‐care program, aimed to determine the effect of telenursing on self‐efficacy and adherence to medication in patients with MI.

**Methods:**

This study was a randomized clinical trial with two intervention and control groups. Sixty‐two patients with MI were randomly assigned. The intervention group received an in‐person session (30–60 min), telephone follow‐up (8 calls in a month), and educational text messages, and the control group received routine care only.

The research tools included the Abbreviated Mental Test (AMT) for cognitive screening and specific questionnaires administered before and 4 weeks after the intervention. Data were analyzed using a paired t‐test, chi‐square, and ANCOVA by SPSS version 26.

**Results:**

At the posttest measurement, results of the ANCOVA showed significantly higher self‐efficacy scores for the intervention group compared to the control group after adjusting for the pretest scores (F_(1,52)_ = 67.31, *p* < 0.001, η^2^_P_ = 0.564). A similar result was obtained for medication adherence (F(1, 52) = 77.39, *p* < 0.001, η^2^_P_ = 0.598). The effect sizes, calculated using partial eta squared, were 0.564 and 0.598, which are considered to be large.

**Conclusion:**

Telenursing based on a self‐care program significantly increases self‐efficacy and medication adherence in MI patients, and it is recommended that telenursing programs be expanded in healthcare systems to promote self‐care and medication adherence.

**Clinical Trial Registration:**

https://irct.behdasht.gov.ir/user/trial/76806/view. IrCTR registry, IRCT20240416061511N1/Registration Date: 2024‐05‐23.

## 1 Background

Myocardial infarction (MI) is one of the most common and severe cardiovascular diseases, which is known as the leading cause of death and disability worldwide [1]. In addition to physical consequences, this disease has profound psychological and social effects on patients and severely reduces their quality of life [2]. In Iran, with rapid changes in the population structure and lifestyle, the prevalence of cardiovascular diseases, including MI, has increased significantly, and This increase, combined with limited access to ccess to specialized nursing services, have revealed the need to use new solutions such as telenursing [3]. This approach can have a significant effect on the management of patients by reducing costs and increasing access to health care. Therefore, this situation reveals the need for continuous and effective management of affected patients [4].

One key factor in the successful management of chronic diseases, including MIs, is self‐care. Self‐care includes patients' behaviors and actions to maintain their health and improve their condition [5]. This process is important in preventing disease complications, reducing frequent hospitalizations, and improving quality of life. However, self‐care is only effective if the patient has a high level of self‐efficacy, meaning that the individual trusts their ability to manage the disease [6].

Self‐efficacy refers to a person's belief in their ability to perform the necessary behaviors to achieve specific goals. This concept plays a fundamental role in managing chronic diseases because the patient's confidence in their ability to deal with the challenges related to the disease is the basis for starting and continuing positive self‐care behaviors [7]. Research has shown that patients with chronic diseases, including cardiovascular diseases, have a high level of self‐efficacy and are more likely to show healthy behaviors, medication adherence, and effective disease management [8, 9].

Medication adherence is also important as a part of the treatment plan for MI patients. Non‐adherence to drug therapy can lead to worsening of symptoms, serious complications, and even death [10]. However, many patients have difficulty adhering to medication due to various reasons, including lack of awareness, psychological problems, or limitations in access to care services [11].

Telenursing, which includes providing nursing services through communication technologies, is considered a new solution to improve self‐efficacy and patient adherence to treatment. By providing the possibility of continuous communication between patients and nurses and receiving the necessary training, telenursing can strengthen the sense of self‐efficacy and promote self‐care behaviors [12]. This method is especially efficient in managing chronic diseases such as MI and brings benefits such as reducing costs, easy access, and improving clinical results [13].

Although telenursing has been used in many countries to manage chronic diseases, in Iran, there is not enough evidence about its effectiveness in patients with MI [14, 15]. Therefore, the present study was designed to fill this gap by investigating telenursing's effect on self‐efficacy and medication adherence in patients with MI based on a self‐care program.

## 2 Materials and Methods

### 2.1 Ethical Approval and Study Design

This article results from a master's thesis in Geriatric Nursing from Guilan University of Medical Sciences, with clinical trial code IRCT20240416061511N1 and ethical code IR.GUMS.REC.1403.032. All ethical considerations were considered, including maintaining confidentiality, voluntary participation in the study, patient confidentiality, unconditional freedom of the patient to withdraw from the study at any stage, etc. Informed consent was obtained to use their data for research purposes.

### 2.2 Type of Study

This study was a randomized clinical trial with two parallel groups (intervention and control) and an allocation ratio of 1:1, conducted in 2024 in Rasht, Iran.

### 2.3 Participants and Study Environment

The research population included elderly patients aged 60 years or older with a confirmed diagnosis of MI hospitalized in the CCU and PCCU of Dr. Heshmat Hospital in Rasht (North of Iran). The inclusion criteria included not having speech and hearing problems, not suffering from psychological diseases, having a landline and cell phone to establish telenursing, willingness to participate in the study, suffering from MI, and having a diagnosis of the disease by a cardiologist. Exclusion criteria included the death of the patient, unwillingness to continue participating, and simultaneous participation in other studies.

### 2.4 Random Assignment Method

The allocation of participants to intervention and control groups was done using block randomization (block sizes of 4 and 6). The allocation list was prepared using the Sealed Envelope Ltd online tool. Allocation concealment was done using opaque and numbered envelopes.

### 2.5 Sample Size

The sample size calculation was done for detecting a difference between the means of two independent samples. With an effect size of 0.8 (Cohen's d) for CSEQ and MMAS‐8 total scores, a power of 0.8, and an alpha value of 0.05, 26 subjects would be required in each group. Assuming a potential drop‐out rate of 15%, 31 subjects were needed in each group.

$$\begin{array}{l} \alpha =0.05\;z_{1-\alpha /2}=z_{0.975}=1.960\\ \beta =0.2\;z_{1-\beta }=z_{0.8}=0.841\\ n=\frac{2{\left(z_{1-\alpha /2}+z_{1-\beta }\right)}^{2}}{d^{2}}+\frac{z_{1-\alpha /2}^{2}}{4}=\frac{2{\left(1.960+0.841\right)}^{2}}{0.8^{2}}+\frac{{\left(1.960\right)}^{2}}{4}=26 \end{array}$$

#### 2.5.1 Study Diagram

**Figure 1:**
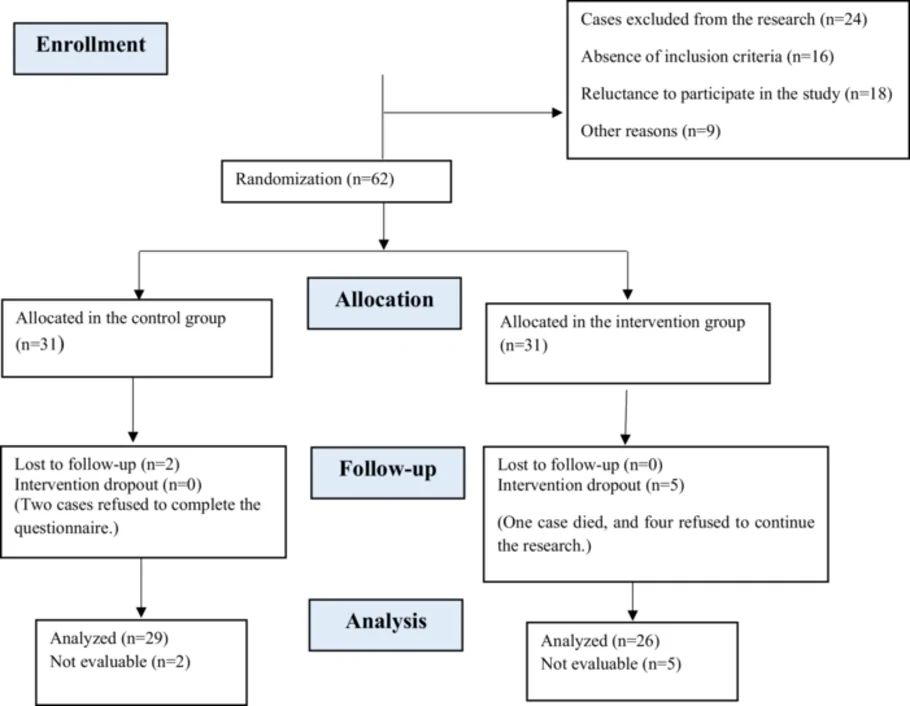
Participant flow diagram.

The CONSORT diagram shows the entry, allocation, and follow‐up process for participants in the control and intervention groups. Sixty‐two patients were randomly enrolled among 120 screened patients, and data from 55 patients were available for the final analysis (Figure 1).

#### 2.5.2 Intervention

The intervention group received a self‐care training program that included the following:

1. In‐person training session: Each individual will be trained separately about heart anatomy, MI pathophysiology, and essential self‐care measures at the hospital. This meeting was held for 30 to 60 min.
2. Telephone follow‐up: Our team made phone calls twice weekly for 1 month after discharge (8 calls total). Each call lasted 10 to 20 min.
3. Messaging follow‐up: During 1 month, 55 educational text messages were sent to the patients of this group to strengthen their learning.

In the control group, there was no intervention, and they only received routine care, including drug recommendations and scheduling for their next doctor's appointments.

#### 2.5.3 Data Collection Method

To conduct the present study, after obtaining permission from the vice president of research, the hospital managers, and the officials of the CCU PCCU of Dr. Heshmat Hospital, the medical records of all patients were reviewed based on the inclusion criteria to determine the eligible patients to participate in the study. Verbal and written consent to participate in the study was obtained from all selected patients who met the inclusion criteria. Initially, the Abbreviated Mental Test (AMT) was administered to assess baseline cognitive status in patients who met the inclusion criteria. The patients were examined in terms of mental and cognitive health, and the ones with an AMT score of 7 or higher were included in the study. The AMT cognitive questionnaire is an abbreviated cognitive test suitable for screening cognitive disorders in the elderly. This questionnaire has 10 items, and the elderly in question must be able to score 7 to 10. A score lower than seven on this test means the presence of cognitive disorders. The items include age, time of day, year, name of residence, identification of two companions or employees, date of birth, year of the Islamic Revolution, name of the leader of the time, ability to count backward from 20 to 1, and repetition of an address. Bakhtiari et al. confirmed this test's validity and reliability with a Cronbach's alpha coefficient of 0.76 [16]. Following cognitive screening, baseline demographics and medical histories were documented. Then, demographic information was taken from the selected patients to determine their demographic information and history of diseases. The demographic questionnaire consists of personal information and information related to the disease. The personal information section included age, gender, place of residence, marital status, level of education, insurance status, employment status, and monthly income. The information related to the disease included questions about the history of other diseases, the duration of the disease, and the duration of treatment. After that, patients were evaluated using Sullivan's Cardiac Self‐Efficacy Questionnaire, and patients with insufficient self‐efficacy (patients who scored between 0 and 22) were included in the study. The cardiac self‐efficacy questionnaire was designed and developed by Sullivan et al. in 1998 to measure cardiac self‐efficacy [17]. This questionnaire consists of 16 questions, the answers scored on a Likert scale from zero (not at all sure) to four (completely sure). In this questionnaire, the total score is between 0 and 64, and higher scores indicate better self‐efficacy. The range of scores of the questionnaire is as follows: scores of 0 to 22 are considered low self‐efficacy (insufficient), scores of 23 to 32 are considered moderate self‐efficacy, and scores of 33 to 64 are considered high self‐efficacy. The validity and reliability of this questionnaire were confirmed by Shamsizadeh et al., and its reliability was assessed as desirable with a Cronbach's alpha coefficient of 0.80 [18]. Then, the study employed a validated questionnaire adapted from the Morisky Medication Adherence Scale (MMAS‐8) for the patients and completed it on their behalf. This questionnaire was designed by Morisky et al. (2008) and was created to measure medication adherence in patients with hypertension [19]. The MMAS questionnaire consists of seven two‐choice questions (with yes and no answers) and one Likert‐type question. Its total score range is between 0 and 8. The higher the score, the higher the medication adherence of the individuals. In questions 1 to 7, yes answers were given a score of 0, and no answers a score of 1. Question 5 was scored in reverse order, and in question 8, the answer “never” was given a score of 0, sometimes a score of 0.25, usually a score of 0.5, usually a score of 0.75, and always a score of 1. The validity and reliability of this questionnaire were confirmed in the study by Yadollahi et al., and its reliability was reported to be 71% using Cronbach's alpha method [19]. Also, in the study by Yang et al., its reliability was calculated to be 72% using Cronbach's alpha test [20].

This study translated the questionnaire into Persian using a forward‐backward translation approach. Two specialists in medical translation and experienced translators of the questionnaire involved in the translation. The translated versions were compared to ensure the questions accurately reflected the intended meanings and concepts. The most appropriate translations were chosen to create a final Persian version of the instrument. To verify that the Persian translation aligned closely with the original text and retained accurate sentence structures, two additional translators fluent in English, who had not seen the original questionnaire, reviewed the initial translation. Two metrics were employed to assess the quantitative content validity. Ihe Content Validity Ratio (CVR) and the Item Content Validity Index (CVI). Initially, the questionnaire was distributed to 12 experts to evaluate the CVR. They rated each item on a three‐point Likert scale, indicating whether it was “useful” or “not necessary.” The CVR was computed using the formula: (ne ‐ (N/2))/(N/2), where N represents the total number of specialists and ne counts those who deemed the item “necessary.” Each expert also evaluated the content validity index (CVI) for each question, providing their ratings. To assess the questionnaire's stability over time (test‐retest), the intra‐class correlation coefficient (ICC) was calculated with a 95% confidence interval. For this assessment, a group of 30 patients completed the tool on two occasions, 2 weeks apart. A score above 0.75 indicates optimal stability. Additionally, the internal consistency of the questionnaire items was determined to be 0.81 using Cronbach's alpha.

Then, the cases were divided into two intervention groups and a control group. In the control group, no intervention was applied. They received routine care that included instructions on taking medications, guidance on activity types, drug recommendations, and scheduling for their next doctor's appointments. For the intervention group, in addition to routine training, a 30–60 min face‐to‐face session was held at the hospital covering the anatomy and physiology of the heart in simple terms, the pathophysiology of MI), and the necessary care and actions for patients to avoid complications. Patients' questions were also answered. After discharge, telephone follow‐up for 1 month was conducted twice a week (8 calls per month) based on the needs of each patient for 10 to 20 min. Then, 55 educational text messages were sent to patients in this group over 1 month to reinforce learning. The educational content of the face‐to‐face sessions and telephone calls was compiled from reputable and relevant books and articles [21, 22]. The content included topics such as anatomy and physiology of the heart, definition of MI, signs, and symptoms of MI, diagnostic tests for MI, self‐care training for MI patients including diet, blood pressure control, blood lipid control, diabetes control, smoking cessation, stress control, adherence to medication regimen, and physical activity. The validity of the educational content was assessed by several nursing professors of the research team, faculty members, and a cardiologist consultant. After receiving their comments, the necessary amendments were made. The patients were provided with a phone number to answer their questions. The subjects were informed about the study's purpose and the confidentiality of the information. Four weeks after the last contact in the intervention group, patients in both groups completed the questionnaires again.

**An example of educational content sent via text messages for the elderly:**

*If you feel very sleepy and cannot stay awake, your family or caregiver must call the doctor or nurse right away*.

*Tell the nurse or doctor if you have any signs of high or low blood sugar*.

*Signs of high blood sugar:*

*Very thirsty, passing urine often, feeling sleepy, feeling shaky, feeling sick (nausea), and blood sugar over 200 mg*.

*Signs of low blood sugar:*

*Feeling irritable or moody, shaking, sweating, feeling dizzy, and having trouble thinking clearly*.

*Learn about the problems that diabetes can cause, their signs, and what you can do to prevent them*.

*Special training:*

*Learn how to check your blood sugar at home by yourself. This is very important. Make sure you have all the equipment you need*.

*Know how often you should check your blood sugar and how to write down the results*.

*Learn:*

- *When to do the test*
- *How to do it correctly*
- *What your target blood sugar range should be*
- *How to read the results?*
- *What to do if the results are not normal*
- *How to clean your equipment?*
- *How to check quality (like checking the expiry date of test strips and getting equipment ready)*

*When you are sick, you need to check your blood sugar more often*.

*If you are ill, check your blood sugar every 4* *hours. Sickness or injury can increase your body's need for sugar. You may also need to change your food or medicines*.

*Test results and signs you must tell the doctor about:*

- *Blood sugar over 300* *mg*
- *Not being able to eat or drink anything by mouth*
- *Any signs of high blood sugar*

*Keep your blood sugar test results near the telephone so you can look at them when you talk to the doctor*.

*To prevent dehydration (not having enough water in your body), drink soup, tea, diet soda, gelatin, and other fluids with electrolytes*.

*Always remember safety and personal hygiene. Pay special attention to your skin, mouth, and teeth*.

*Take good care of your mouth and teeth every day to prevent infection*.

*To protect your feet, wear comfortable shoes at all times, even indoors*.

*Every day, use a hand mirror to check your feet for cuts, scratches, or sores*.

*Keep your skin clean and dry to prevent infection and damage. If you have cold skin, pain, or any sores that do not heal, tell your doctor or nurse right away so they can check you*.

*Wear gloves when gardening*.

*Women should check the genital area for signs of fungal infection. Tell your doctor or nurse if you notice anything unusual*.

*Always carry fast‐acting sugar with you, like candy or sugar tablets, to treat low blood sugar*.

*Get the supplies you may need, such as:*

- *A blood sugar meter*
- *A medical alert bracelet*
- *An ID card*
- *Supportive shoes*

*Talk to your nurse about what you should do when you are planning to travel*.

*Tell your doctor about your travel plans*.

*Keep copies of your prescriptions (medicines, syringes, and needles). Carry a letter that describes your health condition. Get a medical alert bracelet*.

*Plan your meals carefully and eat at regular times*.

*Carry your insulin and equipment in a handbag so they do not get lost and are not exposed to very hot or very cold temperatures*.

*Carry emergency medicines with you in case of problems during travel*.

*Bring enough food with you*.

*Know that when you change time zones, you may need to change your meal times and medicine schedule*.

*Know where you can get emergency help if you need it*.

*If possible, travel with a friend or companion*.

#### 2.5.4 Statistical Analysis

In this study, categorical variables were expressed as frequency (percentage) and continuous variables as mean (standard deviation (SD)). We conducted paired *t* tests to assess changes in CSEQ and MMAS‐8 total scores between pre‐ and post‐test measurement by groups. Additionally, ANCOVA was used to compare the groups at posttest after controlling for pretest scores. The effect size was reported in partial eta squared (η^2^_p_) for ANCOVA and Cohen's d for paired t‐ test; η^2^_p_ values of 0.01–0.06, 0.06–0.14, and > 0.14 and Cohen's d values of 0.2–0.5, 0.5–0.8, and > 0.8 were considered as small, medium, and large effect sizes, respectively. Data analysis was performed using IBM SPSS Statistics for Windows, version 26.0 (IBM Corp., Armonk, NY, USA), and graphs were depicted using GraphPad Prism, Version 8.0.1 (GraphPad Prism Software Inc., San Diego, CA, USA). A *p* < 0.05 was considered statistically significant.

## 3 Results

### 3.1 Patients Characteristics

Figure 1 shows the flow of participants through the trial. A total of 120 patients with MI were screened and 62 patients underwent randomization. Of these, posttest data were available for 55 patients to be included in the intention‐to‐treat analysis (control: 29 patients, and intervention: 26 patients). The demographic and clinical characteristics of the patients are shown in Table 1. The mean age of the patients was 71.6 (SD = 9.1) years and the mean of the disease duration was 6.9 (SD = 6.1) years. Of the patients, 60.0% were female, 49.1% were married, 9.1% had university education, 45.5% were residents in rural areas, and 81.8% had chronic disease. Demographics and clinical characteristics were well balanced between control and intervention groups.

**Table 1:** Personal and clinical characteristics of the patients studied in the control and intervention.

	Total (*n* = 55)	Control group (*n* = 29)	Intervention group (*n* = 26)
	*n* (%)	*n* (%)	*n* (%)
Gender			
Male	22 (40.0%)	12 (41.4%)	10 (38.5%)
Female	33 (60.0%)	17 (58.6%)	16 (61.5%)
Marital status			
Married	27 (49.1%)	15 (51.7%)	12 (46.2%)
Single	3 (5.5%)	1 (3.5%)	2 (7.7%)
Widowed	20 (36.3%)	11 (38.0%)	9 (34.6%)
Divorced	5 (9.1%)	2 (6.8%)	3 (11.5%)
Education			
Illiterate	26 (47.3%)	15 (51.7%)	11 (42.3%)
Below diploma	13 (23.6%)	6 (20.7%)	7 (26.9%)
Diploma	11 (20.0%)	6 (20.7%)	5 (19.2%)
University	5 (9.1%)	2 (6.9%)	3 (11.5%)
Occupation			
Employee	1 (1.8%)	0 (0.0%)	1 (3.8%)
Self‐employed	22 (40.0%)	13 (44.9%)	9 (34.6%)
Retired	15 (27.3%)	7 (24.1%)	8 (30.8%)
Unemployed	17 (30.9%)	9 (31.0%)	8 (30.8%)
Residence			
Urban	30 (54.5%)	15 (51.7%)	15 (57.7%)
Rural	25 (45.5%)	14 (48.3%)	11 (42.3%)
Smoking history			
Yes	21 (38.2%)	12 (41.4%)	9 (34.6%)
No	34 (61.8%)	17 (58.6%)	17 (65.4%)
Chronic disease			
Yes	45 (81.8%)	25 (86.2%)	20 (76.9%)
No	10 (18.2%)	4 (13.8%)	6 (23.1%)
	Mean (SD)	Mean (SD)	Mean (SD)
Age (y)	71.6 (9.1)	70.6 (8.3)	72.7 (10.0)
Disease duration (y)	6.9 (6.1)	7.0 (6.2)	6.9 (6.2)

Abbreviation: SD, Standard Deviation.

### 3.2 Comparison Between Pretest and Posttest Scores

#### 3.2.1 CSEQ Total Score

As presented in Table 2, in the intervention group, the mean of CSEQ total score in the posttest measurement significantly increased by 12.38 (95% CI: 9.57 to 15.20) points compared to the pretest measurement (t_(25)_ = 9.06, *p* < 0.001, Cohen's d = 1.776), while in the control group, the mean of CSEQ total score significantly decreased by 2.31 (4.35, 95% CI: 0.27) points (t_(28)_ = −2.32, *p* = 0.028, Cohen's d = 0.431).

**Table 2:** Within‐group analyses of the CSEQ and MMAS‐8 total scores in patients with MI by control and intervention groups.

	Pretest	Posttest	Mean of differences (95% CI)	t	*p*	Cohen's d
CSEQ total score						
Control	18.00 (8.59)	15.69 (9.36)	−2.31 (−4.35 to −0.27)	−2.32	0.028	0.431
Intervention	23.00 (10.00)	35.38 (14.85)	12.38 (9.57 to 15.20)	9.06	< 0.001	1.776
MMAS‐8 total score						
Control	3.91 (1.04)	3.22 (1.25)	−0.69 (−1.03 to −0.35)	−4.12	< 0.001	0.765
Intervention	3.79 (1.24)	5.60 (1.47)	1.81 (1.32 to 2.30)	7.60	< 0.001	1.489

*Note:* (Figure 2). CI: Confidence Interval; CSEQ: Cardiac Self‐Efficacy Questionnaire; MMAS‐8: 8‐item Morisky Medication Adherence Scale. Cohen's d values of 0.2–0.5, 0.5–0.8, and > 0.8 were considered as small, medium, and large effect sizes, respectively. *p*‐values are based on a paired *t*‐test.

#### 3.2.2 MMAS‐8 Total Score

In the intervention group, the mean of MMAS‐8 total score in the posttest measurement increased significantly by 1.81 (95% CI: 1.32 to 2.30) points compared to the pre‐test measurement (t_(25)_ = 7.60, *p* < 0.001, Cohen's d = 1.489), while in the control group, the mean of MMAS‐8 total scores decreased significantly by 0.69 (95% CI: 0.35 to 1.03) points (*t*_(28)_ = −4.12, *p*< 0.001, Cohen's d = 0.765) (Table 2).

**Figure 2:**
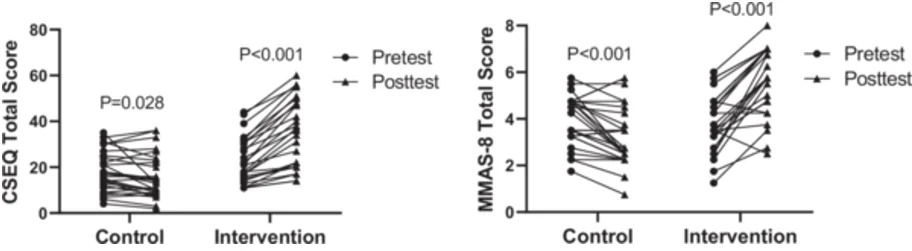
Within‐group analyses of the CSEQ and MMAS‐8 total scores in patients with MI by control and intervention groups. CSEQ, Cardiac Self‐Efficacy Questionnaire; MMAS‐8, 8‐item Morisky Medication Adherence Scale. *p*‐values are based on the paired t‐test.

### 3.3 Comparison Between Control and Intervention Groups

#### 3.3.1 CSEQ Total Score

At the posttest measurement, results of the ANCOVA showed significantly higher CSEQ total scores for the patients in the intervention group compared to the patients in the control group after adjusting for the pretest scores (F_(1,52)_ = 67.31, *p* < 0.001, η^2^_P_ = 0.564). The effect size, calculated using partial eta squared, was 0.564, which is considered to be large (Table 3).

**Table 3:** Evaluating the effect of telenursing on cardiac self‐efficacy and medication adherence in patients with MI.

	Control	Intervention	Adjusted mean difference (95% CI)^a^	F_(1,52)_	*p*	η^2^_p_
CSEQ total score						
Pretest	18.00 (8.59)	23.00 (10.00)				
Posttest	15.69 (9.36)	35.38 (14.85)	13.94 (10.53 to 17.35)	67.31	< 0.001	0.564
MMAS‐8 total score						
Pretest	3.91 (1.04)	3.79 (1.24)				
Posttest	3.22 (1.25)	5.60 (1.47)	2.47 (1.91 to 3.04)	77.39	< 0.001	0.598

*Note:* (Figure 3). CI: Confidence Interval; CSEQ: Cardiac Self‐Efficacy Questionnaire; MMAS‐8: 8‐item Morisky Medication Adherence Scale. Data are mean (SD), unless otherwise specified. η^2^_p_ values of 0.01–0.06, 0.06–0.14, and > 0.14 were considered as small, medium, and large effect sizes, respectively.

^a^ Adjusted for pretest scores.

#### 3.3.2 MMAS‐8 Total Score

After adjusting for pretest scores, patients in the intervention group scored, on average, 2.47 (95% CI: 1.91 to 3.04) points higher on the MMAS‐8 total score than patients in the control group at the posttest assessment (F_(1,52)_ = 77.39, *p* < 0.001, η^2^_P_ = 0.598). The effect size was 0.598, which is considered to be large (Table 3).

**Figure 3:**
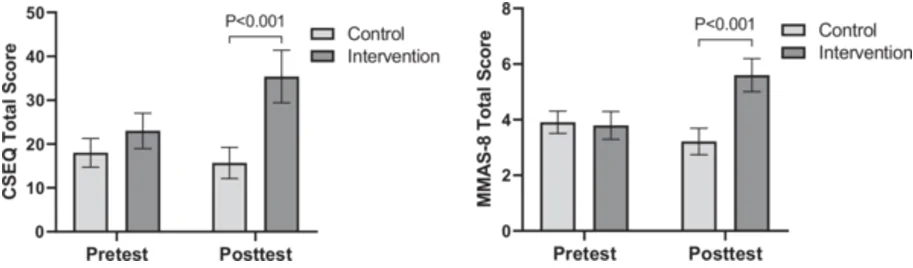
Evaluating the effect of telenursing on cardiac self‐efficacy and medication adherence in patients with MI. CSEQ, Cardiac Self‐Efficacy Questionnaire; MMAS‐8, 8‐item Morisky Medication Adherence Scale. Values are presented the mean with a 95% confidence interval. Between‐group differences were examined using ANCOVA after adjusting for pretest scores.

## 4 Discussion

This study was conducted to determine the effect of telenursing based on a self‐care program on self‐efficacy and adherence to medication in patients with MI. The findings of the present study regarding the comparison of the mean self‐efficacy scores before and after the intervention between the intervention and control groups showed that the self‐efficacy score in the intervention group was significantly higher than that in the control group. This highlights that systematic telephone follow‐up paired with remote health education yields a robust clinical impact on patient self‐care dynamics. In line with this finding, the findings of the study by Keshavarz et al. [23], Gohari et al. [24], and Behzad et al. [25] showed that education in methods such as face‐to‐face education followed by remote follow‐up and telephone follow‐up could improve the self‐efficacy of patients in the intervention group compared to the control group, because telephone follow‐up can increase patients' self‐efficacy regardless of time and place, creating and maintaining a dynamic, flexible, and continuous care relationship between the nurse and the patient.

However, in contrast to this finding, Lee et al. reported that the level of self‐efficacy among hemodialysis patients in the post‐intervention stage did not differ significantly from that of the control group [26]. The difference in the study findings can be attributed to the different types of patients studied because hemodialysis patients can experience numerous other problems due to the chronic nature of the disease, which can affect the self‐efficacy of these patients. The present study's results are also inconsistent with the study by Aimi et al. [27]. The findings of their study showed that multimedia and virtual education did not affect the level of self‐efficacy of patients with chronic pulmonary obstruction. It seems that the difference in the findings of the two present studies is related to the type of educational content and the type of underlying disease of the patients. In that study, researchers were trained on medical equipment and how to use it, and it is expected that the study results will change due to the different levels of education and understanding of the patients. In addition, patients with respiratory problems due to pulmonary problems have lower levels of activity tolerance and training than the patients in the present study, and this factor could also be one of the reasons for the differences in the results of the two studies.

The present study's findings regarding comparing the mean scores of medication adherence before and after the intervention between the intervention and control groups showed that the mean scores of medication adherence in the intervention group increased after the intervention compared to the control group, which was statistically significant. This means the intervention group had better medication adherence than the control group. This finding from the present study was consistent with the study by Park et al. [28]. The study's findings by Park et al. showed that using educational support tools and telephone reminders significantly increased patients' medication adherence compared to the control group. The findings of the study by Kamrani et al. are consistent with the present study's findings, showing that adherence to the treatment regimen increased more in the telephone follow‐up and education groups compared to the control group [15].

However, this present study's finding was contrary to the study by Gamar et al. The findings of their study showed that in the medication adherence dimension, education in two methods with and without telephone follow‐up had no significant effect on increasing medication adherence in patients with MI compared to the control group [29]. Methodological differences likely explain this conflict. In the present study, the cognitive level of the patients was first examined, and patients with a cognitive level higher than seven were included as the inclusion criteria. Then, patients with a level of self‐efficacy were identified, and these cases were considered after the two of intervention. However, these cases were not considered in the study of those researchers. Also, the difference in the education content, measurement tool, and 1‐month messaging follow‐up could be other reasons for the inconsistency of the results.

On the other hand, different from the present findings, the study by Bersing et al. showed no statistically significant difference between the two study groups regarding medication adherence [30]. The reason for the inconsistency of the results can be attributed to the difference in the type of disease. Rheumatoid arthritis patients have chronic and persistent pain. Reduced mobility and dependence on others are other characteristics of these patients. Therefore, the patient's inability to perform personal tasks and dependence on others is perhaps the most important reason for the difference in the findings.

The findings of this study indicated the positive effect of telenursing based on a self‐care program on improving self‐efficacy and adherence to medication in patients with MI. These results emphasize the importance of modern communication technologies in providing nursing services, which can be used as an effective strategy for managing chronic diseases. From a clinical perspective, telenursing can facilitate patients' access to education and follow‐up care by reducing geographical and time constraints and improving their treatment outcomes. This is especially important in countries with developing health systems, such as Iran, which may face limitations in providing nursing services.

## 5 Conclusion

Telenursing based on a self‐care program is a practical approach to improving self‐efficacy and adherence to medication in patients with MI. Creating continuous and dynamic communication between the patient and the nurse can reduce barriers to access to care services and improve the quality of chronic disease management. Telenursing can be used in health systems, especially in conditions of resource and access constraints, as a practical and cost‐effective solution. Expanding this intervention and implementing it in other patient groups paves the way for further research and improving the performance of health systems. Telenursing can bridge gaps in healthcare access, especially in developing regions like Iran, thereby improving patient outcomes and the overall quality of care. Future research should continue to explore the long‐term effects of telenursing interventions and their applicability across diverse patient populations and health conditions.

## 6 Clinical Application

The findings of this study can provide practical guidance for nurses in enhancing their role in remote care. Telenursing allows nurses to provide educational and supportive services to patients in less time and facilitates ongoing follow‐up. For hospital administrators, these results represent a cost‐effective strategy to reduce the burden of repeated hospitalizations and increase patient satisfaction. Policymakers can also use this evidence to develop national telenursing programs and improve access to health services in underserved areas.

## 7 Limitations and Strengths

The strengths of this study include the regular follow‐up of patients through telephone calls and sending educational text messages, as continuous communication with patients has enhanced the effects of the intervention. The findings of the present study provide a comprehensive source for the implementation of telenursing in the healthcare setting. In addition, attention to patients' individual needs through personalization of education and answering their specific questions in telephone calls has increased the effects of the intervention. However, the study also had limitations. One limitation is the reliance on patient self‐reported data, which may introduce bias. To address the limitation of relying on patient self‐reported data and to minimize potential bias allow for anonymous reporting when possible, encouraging patients to be more honest about sensitive topics, which can lead to more accurate data. In addition, factors such as social support or economic status, which could significantly impact the results, were not examined in the study. Also, Follow‐up duration was only 4 weeks, with no evaluation of long‐term effects, This was a single‐center study with limited sample representativeness, The impact of the intervention on clinical outcomes was not evaluated, with only a focus on intermediate outcome indicators.

Corresponding future research directions: Coduct multi‐center and long‐term follow‐up studies, and include confounding factors such as social factors.

## Author Contributions

**Arian Mobasheri:** conceptualization, investigation, writing – original draft, methodology, visualization. **Parand Pourghane:** conceptualization, methodology, supervision, visualization, project administration, writing – review and editing. **Fereshteh Besharati:** conceptualization, methodology, validation, writing – original draft. **Saman Maroufizadeh:** conceptualization, methodology, software, data curation, formal analysis.

## Funding

The authors have nothing to report.

## Disclosure

All authors have read and approved the final version of the manuscript corresponding author Dr. Parand Pourghane had full access to all of the data in this study and takes complete responsibility for the integrity of the data and the accuracy of the data analysis.

## Ethics Statement

This article results from a master's thesis in Geriatric Nursing from Guilan University of Medical Sciences, with clinical trial code IRCT20240416061511N1 and ethical code IR.GUMS.REC.1403.032. The studies were conducted in accordance with the local legislation and institutional requirements. The current study included only those who presented their informed consent. For this purpose, an informed consent form was completed by all participants after being informed of the study's aims. All participant data were de‐identified and kept anonymous; no personal identifiers that could link the answers with any of the participants in the present study. All methods in the study were in accordance with relevant regulations & guidelines (General Ethical Guidance for Medical Research with Human Participants in the Islamic Republic of Iran). All authors have read and approved the final version of the manuscript. Dr. Parand Pourghane had full access to all of the data in this study and takes complete responsibility for the integrity of the data and the accuracy of thedata analysis. This manuscript has not been previously published. The corresponding author (Dr. Parand Pourghane) affirms that this manuscript is an honest, accurate, and transparent account of the study have been omitted, and that any discrepancies from the study as planned have been explained.

## Conflicts of Interest

The authors declare no conflicts of interest.

## Transparency Statement

The authors affirm that the design, implementation, analysis, and reporting of this randomized clinical trial were conducted with full transparency and adherence to ethical research standards. All procedures—including patient recruitment, randomization, intervention delivery, and data collection—were performed according to the approved study protocol.

## Data Availability

The data that support the findings of this study are available from the corresponding author upon reasonable request. The datasets used and/or analyzed during the current study are available from the corresponding author on reasonable request. The leadauthor affirms that this manuscript is anhonest, accurate, and transparentaccount of the study beingreported; that no importantaspects of the study have beenomitted; and that anydiscrepancies from the study asplanned have been explained.

## References

[1] F. Rodrigues , C. Domingos , D. Monteiro , and P. Morouço , “A Review on Aging, Sarcopenia, Falls, and Resistance Training in Community‐Dwelling Older Adults,” International Journal of Environmental Research and Public Health 19, no. 2 (2022): 874, https://pubmed.ncbi.nlm.nih.gov. 35055695 10.3390/ijerph19020874 PMC8775372

[2] S. Cowley , V. Tzouvara , and T. H. R. Da Silva , “Public Health: Healthy Ageing and Well‐Being,” Redfern's Nursing Older People 87 (2023): 1, https://kclpure.kcl.ac.uk.

[3] J. Dindar Farkosh , S. Kazemipour Sabet , and H. Ansari , “Foresight of the Aging Trend of the Iranian Population in Different Regions and Population Groups Until 1420,” Future Study Management. 33 (2022): 1401, 10.30495/jmfr.2022.20259.

[4] L. Mamashli , “Telenursing in Cardiovascular Diseases: A Critical Review of Systematic Reviews of Evidence,” Iranian Journal of Systematic Review in Medical Sciences 1, no. 3 (2023): 20–32, http://ijsr.ir/article-1-74-en.html.

[5] H. Mahmoudzadeh , T. Aghayari Hir , and D. Hatami , “Study and Analysis of the Elderly Population of the Iran,” Geographical Researches 37, no. 1 (2022): 111–125, 10.29252/geores.37.1.111.

[6] R. Novrouzi , M. Ghaffari , M. Sabouri , T. Marashi , and S. Rakhshanderou , “Investigating the Effect of Self‐Care on the Nutritional Status of the Elderly by Structural Equation Modeling Analysis,” Iranian Journal of Ageing 18, no. 1 (2023): 46–59, 10.32598/sija.2022.3368.1.

[7] S. W.‐C. Chan , “Chronic Disease Management, Self‐Efficacy and Quality of Life,” Journal of Nursing Research 29, no. 1 (2021): e129, 10.1097/JNR.0000000000000422. PMC7808345 33427791

[8] T. T. H. Dinh and A. Bonner , “Exploring the Relationships Between Health Literacy, Social Support, Self‐Efficacy and Self‐Management in Adults With Multiple Chronic Diseases,” BMC Health Services Research 23, no. 1 (2023): 923, 10.1186/s12913-023-09907-5. 37649013 PMC10466814

[9] H. Farley , “Promoting Selfefficacy in Patients With Chronic Disease Beyond Traditional Education: A Literature Review,” Nursing Open 7, no. 1 (2020): 30–41, 10.1002/nop2.382. 31871689 PMC6917929

[10] J. Redfern , Q. Tu , K. Hyun , et al., “Mobile Phone Text Messaging for Medication Adherence in Secondary Prevention of Cardiovascular Disease,” Cochrane Database of Systematic Reviews 2024, no. 3 (2024): CD011851, 10.1002/14651858.CD011851.pub3. PMC10966941 38533994

[11] P. Natarajan , “Genomic Aging, Clonal Hematopoiesis, and Cardiovascular Disease,” Arteriosclerosis, Thrombosis, and Vascular Biology 43, no. 1 (2023): 3–14, 10.1161/ATVBAHA.122.318181. 36353993 PMC9780188

[12] M. Sasanipour and A. Khosravi , “Increase in Life Expectancy Due to Changes in the Patterns of Deaths from Cardiovascular Diseases in Iran During 2006–2019,” Payesh (Health Monitor) 21, no. 6 (2022): 581–592, 10.52547/payesh.21.6.581.

[13] X. Qiu , “Nurse‐Led Intervention in the Management of Patients With Cardiovascular Diseases: A Brief Literature Review,” BMC Nursing 23, no. 1 (2024): 6, 10.1186/s12912-023-01422-6. 38163878 PMC10759353

[14] P. Fathizadeh , H. Heidari , R. Masoudi , M. Sedehi , and F. Khajeali , “Telenursing Strategies in Iran: A Narrative Literature Review,” International Journal of Epidemiology and Health Sciences 1, no. e03 (2020): 1–15, 10.51757/IJEHS.1.3.2020.46189.

[15] F. Kamrani , S. Nikkhah , F. Borhani , M. Jalali , S. Shahsavari , and K. Nirumand‐Zandi , “The Effect of Patient Education and Nurse‐Led Telephone Follow‐up (Telenursing) on Adherence to Treatment in Patients With Acute Coronary Syndrome,” Cardiovascular Nursing Journal 4, no. 3 (2015): 16–24, http://journal.icns.org.ir/article-1-346-en.html.

[16] F. Bakhtiyari , M. Foroughan , H. Fakhrzadeh , et al., “Validation of the Persian Version of Abbreviated Mental Test (AMT) in Elderly Residents of Kahrizak Charity Foundation,” Iranian Journal of Diabetes and Metabolism 13, no. 6 (2014): 487–494, http://ijdld.tums.ac.ir/article-1-5271-en.html.

[17] M. D. Sullivan , A. Z. LaCroix , J. Russo , and W. J. Katon , “Self‐Efficacy and Self‐Reported Functional Status in Coronary Heart Disease: A Six‐Month Prospective Study,” Psychosomatic Medicine 60, no. 4 (1998): 473–478, 10.1097/00006842-199807000-00014. 9710293

[18] M. Shamsizadeh , S. Shaadi , Y. Mohammadi , and S. R. Borzou , “The Effects of Education and Telephone Nurse Follow‐Up (Tele‐Nursing) on Diabestes Management Self–Efficacy in Patients With Type 2 Diabetic Referred to Hamadans Diabetes Center in (2018),” Avicenna Journal of Nursing and Midwifery Care 29, no. 2 (2021): 81–90, 10.30699/ajnmc.29.2.81.

[19] S. Yadollahi , T. Ashktorab , and F. Zayeri , “Medication Adherence and Related Factors in Patients With Epilepsy,” Hayat, Journal of School of Nursing and Midwifery 21, no. 2 (2015): 67–80, http://hayat.tums.ac.ir/article-1-1135-en.html.

[20] A. Yang , B. Wang , G. Zhu , et al., “Validation of Chinese Version of the Morisky Medication Adherence Scale in Patients With Epilepsy,” Seizure 23, no. 4 (2014): 295–299, 10.1016/j.seizure.2014.01.003. 24484672

[21] S. Sharafi , T. Nasrabadi , A. Beyranvand , and A. Sheykhi , Self‐Care in Coronary Artery Diseases (Tehran: Jame_e Negar Publishing House, 2017), https://basalam.com.

[22] S. Kumar , The Power of Self‐Care: Transforming Heart Health With Lifestyle Medicine (India: Notion Press, 2023). Power‐Self‐Care‐Transformin, https://www.amazon.in.

[23] N. Keshavaraz , M. Naderifar , M. Firouzkohi , A. Abdollahimohammad , and M. R. Akbarizadeh , “Effect of Telenursing on the Self‐Efficacy of Patients With MI: A Quasi‐Experimental Study,” Signa Vitae 16, no. 2 (2020): 92–96, 10.22514/sv.2020.16.0039.

[24] F. Gohari , S. Hasanvand , M. Gholami , H. Heidari , and P. Baharvand , “Effectiveness of Rehabilitation by Telephone Follow‐Up on the Self‐Efficacy of Patients Undergoing Coronary Artery Bypass Graft Surgery,” Interdisciplinary Journal of Acute Care 1, no. 2 (2021): 66–73, 10.22087/ijac.2020.127194.

[25] Y. Behzad , F. Bastani , and H. Haghani , “Effect of Empowerment Program With the Telephone Follow‐Up (Tele‐Nursing) on Self‐Efficacy in Self‐Care Behaviors in Hypertensive Older Adults,” Nursing and Midwifery Journal 13, no. 11 (2016): 1004–1015, http://unmf.umsu.ac.ir/article-1-2422-en.html.

[26] M. C. Lee , S. F. V. Wu , K. C. Lu , C. Y. Liu , S. Y. Liang , and Y. H. Chuang , “Effectiveness of a Selfmanagement Program in Enhancing Quality of Life, Selfcare, and Selfefficacy in Patients With Hemodialysis: A Quasiexperimental Design,” Seminars in Dialysis 34, no. 4 (2021): 292–299, 10.1111/sdi.12957. 33533048

[27] C. Emme , E. L. Mortensen , S. Rydahl‐Hansen , et al., “The Impact of Virtual Admission on Self‐Efficacy in Patients With Chronic Obstructive Pulmonary Disease—A Randomised Clinical Trial,” Journal of Clinical Nursing 23, no. 21–22 (2014): 3124–3137, 10.1111/jocn.12553. 24476457

[28] L. G. Park , J. Howie‐Esquivel , M. L. Chung , and K. Dracup , “A Text Messaging Intervention to Promote Medication Adherence for Patients With Coronary Heart Disease: A Randomized Controlled Trial,” Patient Education and Counseling 94, no. 2 (2014): 261–268, 10.1016/j.pec.2013.10.027. 24321403

[29] E. Gomar , A. Karampourian , M. M. Vardanjani , B. Manafi , and S. Khazaei , “Effect of Education and Telephone Follow‐Up on Patients' Adherence to the Treatment Regimen After Myocardial Infarction,” Avicenna Journal of Nursing and Midwifery Care 30, no. 3 (2022): 151–162, 10.32592/ajnmc.30.3.151.

[30] J. A. Unk and R. Brasington , “Efficacy Study of Multimedia Rheumatoid Arthritis Patient Education Program,” Journal of the American Association of Nurse Practitioners 26, no. 7 (2014): 370–377, 10.1002/2327-6924.12064. 24170559

